# A low proportion of malnourished patients receive nutrition treatment — results from nutritionDay

**DOI:** 10.1080/16546628.2017.1391667

**Published:** 2017-10-25

**Authors:** C. Henriksen, I. M. Gjelstad, H. Nilssen, R. Blomhoff

**Affiliations:** ^a^ Department of Nutrition, Faculty of Medicine, University of Oslo, Oslo, Norway; ^b^ Division of Cancer Medicine, Oslo University Hospital, Oslo, Norway; ^c^ Department of Clinical Nutrition, University Hospital of North Norway, Tromsø, Norway

**Keywords:** Malnutrition, nutritionDay, food intake, BMI, weight loss

## Abstract

NutritionDay is a yearly point-prevalence study of malnutrition in hospitals from more than 50 countries. The aim of the present study was to quantify the frequency of malnutrition and the proportion of malnourished patients receiving nutritional treatment in two university hospitals in Norway using data from nutritionDay. All units at Oslo University Hospital (OUH) and University Hospital of Northern Norway (UNN) were invited to participate in nutritionDay 2014, and 28 out of 85 eligible units agreed to take part. Malnutrition was diagnosed based on body mass index (BMI), weight reduction and food intake in the previous week, according to national guidelines and ESPEN criteria. Data from 488 patients were available, representing 90.1% of occupied beds in participating units. Thirty percent of the patients were diagnosed malnourished when national criteria were used, and only 41% of these patients received nutritional treatment. The estimated malnutrition rate was 11% when the ESPEN consensus criteria were used. Data on weight or height were frequently missing in the patient records, and BMI could only be calculated in two-thirds of the patients. The frequency of low BMI (<18.5 kg/m^2^) was only 5%. Involuntary weight loss was present in 37% of the patients, and 60% had eaten less than normal in the previous week. Oncology units had the highest frequency of patients with low BMI, and the highest weight loss and overall malnutrition rate. Surgery and geriatric units had the highest rate of patients with low food intake. In this study, nearly 60% of the malnourished patients did not receive any nutritional treatment, and this indicates a potential for improved nutritional care and cost savings. Low food intake and weight loss were frequent at these two Norwegian hospitals, and in line with previous reports from nutritionDay in other countries.

## Introduction

NutritionDay is a yearly point-prevalence study of malnutrition in hospitals from more than 50 countries [1]. Malnutrition is defined as a state resulting from lack of nutrition, leading to altered body composition, function and impaired clinical outcome [2]. Several screening tools and diagnostic criteria exist, but there is no general consensus on which are preferable. The prevalence of malnutrition in European hospitals ranges from 20 to 60% [3–6]. The wide range may be explained by regional variations and use of different tools and criteria. Earlier results from nutritionDay indicate that 27% of the patients are at nutritional risk worldwide [7].

Being malnourished or at nutritional risk is associated with higher morbidity, longer stay in hospital and increased mortality [8], leading to personal and economic burden for the patient and society. A screening survey of more than 500,000 patients from the Netherlands showed that patients diagnosed with malnutrition stayed in hospital 1.4 days longer compared to those who were well nourished [9]. A Spanish study estimated the increased cost for each malnourished patient to be 45–105% [10]. The possible savings of identifying and treating malnourished patients in Norwegian hospitals have earlier been estimated to be in the range of 30–100 million euros per year, assuming that 30% of the patients are discharged one day earlier because of correct treatment [11,12], but the numbers are uncertain and updated calculations are needed.

It is crucial to identify patients at risk and correctly diagnose those who are malnourished. In Norway it is recommended that all patients are screened for nutritional risk at admittance and weekly with one of the following screening tools: NRS-2002, MUST or MNA [13]. Using the NRS-2002 screening tool, one-third of the patients in the western part of Norway were at nutritional risk or malnourished [14]. The estimate is even higher among older patients; Eide et al. found that 45% of patients older than 70 years were at nutritional risk [15].

Not all patients at risk of malnutrition are actually malnourished, and further assessment is needed for an accurate diagnosis. National guidelines for using the ICD-10 codes for moderate or severe malnutrition are based on specific cut-off points for body mass index (BMI), weight loss and food intake in the previous week [13]. Recently the ESPEN consensus criteria were published diagnosing malnutrition based on BMI <18.5 kg/m^2^ (alternative 1), or specific cut-off points for BMI, weight loss in combination with reduced BMI/fat free mass index (alternative 2), see Table 1 [2].

**Table 1.** Diagnostic criteria of malnutrition.

	National malnutrition criteria^a^	ESPEN malnutrition criteria
1	BMI < 18.5 kg/m^2^^b^	BMI <18.5 kg/m^2^
2	Weight loss > 10% last 3 months	Weight loss > 10% indefinite of time and BMI < 20 kg/m^2^^c^ or low fat free mass
3	Weight loss > 5% last 3 months and BMI < 20 kg/m^2^^c^	Weight loss > 5% in the last 3 months and BMI < 20 kg/m^2^^c^ or low fat free mass
4	Low food intake < 50% last week	

^a^Adapted to the time frame used in nutritionDay

^b^20 kg/m^2^ for persons > 70 years

^c^22 kg/m^2^ for persons > 70 years

Earlier studies have assessed nutritional risk using data from a screening tool and not estimated the malnutrition rate directly [14–17]. We are not aware of any studies from hospitals in the Nordic countries where the actual malnutrition rate has been estimated using the recommended national criteria.

The aim of the present study was to assess the frequency of malnutrition and the number of malnourished patients receiving advanced nutritional therapy, in two different university hospitals in Norway using data from nutritionDay 2014. Secondly we aimed to estimate the potential for cost savings from preventing and treating malnutrition.

## Subject and methods

### Design

NutritionDay is a worldwide project with the aim to improve knowledge and awareness of malnutrition in hospitals. It is a one-day annual cross-sectional study with one-month follow-up using standardized questionnaires. Hospital staff fill in two questionnaires: the hospital sheet asks for nutrition related organisational and structural information on the ward. This questionnaire has to be completed once per ward (Form 1). For each patient, a nurse fills in a patient sheet including data on patient’s characteristics, body weight, height and nutritional therapy (Form 2). The data were collected from the medical records. In addition, each patient is asked to fill in two questionnaires about the amount of food eaten in the previous week and on nutritionDay, the reasons for decreased nutritional intake and changes in body weight during the last three months (Forms 3a/b). The patients had help from a nurse or trained dietitian student to fill in the form if needed. A detailed description of the study design and its main outcomes has been published [18].

The current study includes the two university hospitals in Norway participating in November 2014: Oslo University Hospital (OUH) and University Hospital of Northern Norway (UNN). All somatic units, except delivery and maternity units were invited to participate via an email to the head nurse from the Nutrition Board at the hospitals. There were 22 out of 72 (30%) units participating in OUH, including four paediatric units and two intensive care units. At UNN, six out of 13 units (45%) agreed to participate. Anonymous data were uploaded on the NutritionDay online database.

Data on anthropometry and prevalence of malnutrition are presented for all adult patients aged 18 years or more. BMI was calculated based on height and weight collected from the medical records (weight (kg)/height (m)^2^). Data on weight loss in the last three months (rounded to the nearest 2 kg) were collected from the self-reporting questionnaires together with data on food intake in the previous week (assessed using the following categories: normal, a bit less than normal, less than half of normal and less than quarter to nearly nothing). Food intake was based on the recorded food intake at one of the main meals on nutritionDay (meal consumption was divided as follows: all, half, quarter and nothing). Data on nutrition therapy were collected from the medical records. Nutritional therapy was categorized as follows: enteral nutrition, parenteral nutrition, enteral + parenteral nutrition, protein/energy supplement. Regular hospital food or special diets (e.g. for food allergy) were not regarded as nutrition therapy.

Malnutrition was defined according to the Norwegian guidelines for prevention and treatment of disease related malnutrition [8], as well as the ESPEN consensus definition [2], Table 1.

In addition, the national diagnostic criteria include the subterm: *serious malnutrition* if BMI is less than 16 kg/m^2^ or <18.5 kg/m^2^ in patients over 70 years) *or* weight loss is more than 15% within the last 3 months *or* combination of low BMI (<18.5 kg/m^2^, <20 kg/m^2^ in patients over 70 years) and weight loss more than 5 % the last 3 months *or* food intake in the previous week is less than 25% of estimated need [8].

### Statistics

Results from the two hospitals in different regions were reported separately, as well as for the different specialties. Data are presented as means with standard deviations or number and percentages. Comparisons between hospitals are conducted with Chi-square test for categorical variables and *t*-test for continuous variables. Statistical significance was set at *p* < 0.05, and all analyses were carried out by IBM SPSS Statistics V22.0.

### Ethics

The study was conducted according to the Helsinki Declaration. Because of the non-invasive design, and since the main purpose was a quality improvement, the study was exempted from review by the Regional Committee for Research Ethics. The hospitals Data Protection Official approved the study and the procedures for informed consent. The participants in OUH gave written informed consent, whereas the participants in UNN gave verbal informed consent after receiving written and verbal study information, according to local differences in procedures.

## Results

### Patient characteristics

Data from 488 patients were available, representing 90.1% of occupied beds in participating units. The patients were admitted to units covering a range of specialties, with surgery, internal medicine, oncology and orthopaedic being the most frequent (Table 2). Only data from participants ≥18 years are presented in the further analysis (*n *= 437).

**Table 2.** Subject characteristics in the two Norwegian centres.

	Total *n*=488	OUH *n*=365	UNN *n*=123
Total beds in participating units (*n*)	593	457	136
Beds per unit (mean, SD)	22.8 ± 7.9	22.9 ± 8.5	22.7 ± 5.7
Occupied beds (*n*)	538	413	125
Participation rate of occupied			
beds (%)	90.1	88.4	98.4
Specialty (*n*, %):			
– Internal medicine	93 (19.1)	53 (14.5)	40 (32.5)
– Surgery	163 (33.4)	139 (38.1)	24 (19.5)
– Oncology	46 (9.4)	20 (5.5)	26 (21.1)
– Geriatric	12 (2.5)	0	12 (9.8)
– Paediatric	50 (10.2)	50 (13.7)	0
– Orthopaedic	43 (8.8)	43 (11.8)	0
– Other	81 (16.6)	60 (16.4)	21 (17.1)
Sex (Female/male, %)	50/50	50.4/49.6	51.2/48.8
Age (mean, SD)			
– < 18 years, *n*=51	13 ± 3	13 ± 3	
– 18-69 years, *n*=272	51 ± 14	50 ± 14	54 ± 13
– ≥70 years, *n*=164	79 ± 7	79 ± 7	80 ± 7

OUH: Oslo University Hospital; UNN: University Hospital of North Norway; SD: standard deviation.

### Malnutrition rate

#### Weight and BMI

**Figure 1.**
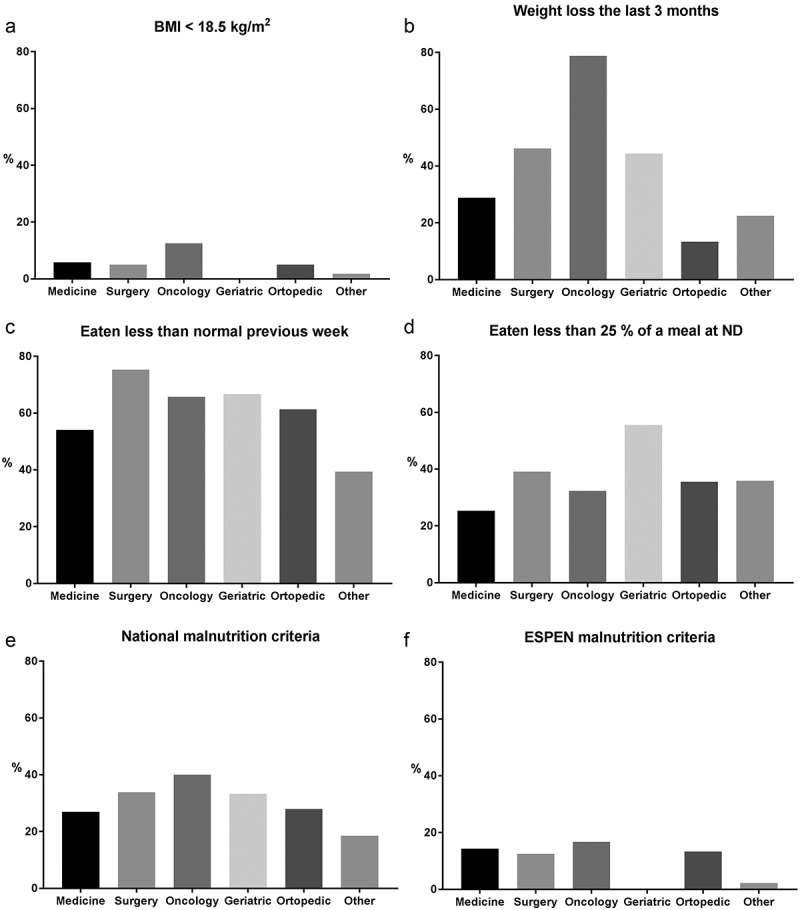
(a-f) Proportion of patients with malnutrition in different specialty units (*n *= 437).

According to the national procedure for nutritionDay, the most recent body weight recorded in the medical record during the previous week was registered, and 75% of the patients had data on body weight. Data on height were only registered in 70% of the patients, so BMI could only be calculated for 66% of the patients. The overall frequency of low BMI (<18.5 kg/m^2^) was 5% and varied from none to 12.5%, where the oncology unit had the highest frequency (Table 3, Figure 1). The weight and frequency of low BMI was not significantly different between the hospitals, but the BMI was lower in OUH compared to UNN (Table 3).

**Table 3.** Anthropometry and weight loss in adult patients.

	Total *n*=437	OUH *n*=315	UNN *n*=122	p-value
Weight registered (*n*)	317	209	108	
Weight in kg (mean, SD)	75.2 ± 17.2	75.0 ± 16.5	75.5 ± 18.7	ns
Height registered (*n*)	307	202	105	
Height in m (mean, SD)	1.71 ± 0.10	1.73 ± 0.10	1.68 ± 0.10	<0.001
BMI calculated (*n*)	290	186	105	
BMI in kg/m^2^ (mean, SD)	25.7 ± 5.5	25.9 ± 4.7	26.7 ± 6.7	0.013
BMI category (n, %)				
Total (*n*)	290	186	104	
≥22	222 (76.6)	141 (75.8)	81 (77.9)	
20-21.9 kg/m^2^	34 (11.7)	19 (10.2)	15 (14.4)	ns
18.5-19.9 kg/m^2^	19 (6.6)	15 (8.1)	4 (3.8)	
16.1-18.4 kg/m^2^	9 (3.1)	7 (3.8)	2 (1.9)	
≤16 kg/m^2^	6 (2.1)	4 (2.2)	2 (1.9)	
Involuntary weight loss (*n*, %)				
Total (*n*)	316	215	101	
Yes	118 (37.3)	84 (39.1)	34 (33.7)	ns
No	186 (58.9)	126 (58.6)	60 (59.4)	
Don’t know	12 (3.8)	5 (2.3)	7 (6.9)	
If yes, % weight loss (*n*, %)				
< 5%	25 (18.2)	18 (21.4)	7 (20.6)	ns
5-10%	21 (17.8)	18 (21.4)	3 (8.8)	
>10%	38 (32.2)	27 (32.1)	11 (32.4)	
Don´t know	34 (28.8)	21 (25.0)	13 (38.2)	
Food intake last week (*n*, %)				
Total (*n*)	311	215	96	
Normal	124 (39.9)	80 (37.2)	44 (45.8)	ns
A bit less than normal	90 (28.9)	61 (28.4)	29 (30.2)	
Less than 50% of normal	38 (12.2)	26 (12.1)	12 (12.5)	
Less than 25% of normal	59 (19.0)	48 (22.3)	11 (11.5)	
Amount eaten at a meal at				
NutritionDay (*n*, %)				
Total (*n*)	294	201	93	
Everything	135 (45.9)	86 (42.8)	49 (52.7)	ns
About 50%	57 (19.4)	41 (20.4)	16 (17.2)	
About 25%	31 (10.5)	23 (11.4)	8 (8.6)	
Nothing	71 (24.1)	51 (25.4)	20 (21.5)	

OUH: Oslo University Hospital; UNN: University Hospital of North Norway; SD: standard deviation.

#### Weight loss

Self-reported involuntary weight loss was present in 37% of the patients, varying from 13 to 79% between specialties, again the oncology units having the highest frequency (Table 3, Figure 1). However, nearly one-third of the patients did not know how much weight they had lost. No significant differences were detected between the hospitals.

#### Food intake

As many as 60% of the patients reported that they had eaten less than normal during the previous week (Table 3). The proportion varied from 39 to 75% between the units, surgery units having the highest frequency (Figure 1). No significant differences were detected between the hospitals.

Less than half of the patients reported that they had eaten the whole meal (lunch or dinner) offered at the time of the survey (Table 3, Figure 1). As many as 35% of the patients ate a quarter or less of the meal, ranging from 25 to 56% between the units, where geriatric units had the lowest food intake. No significant differences were detected between the hospitals.

#### Malnutrition

**Figure 2.**
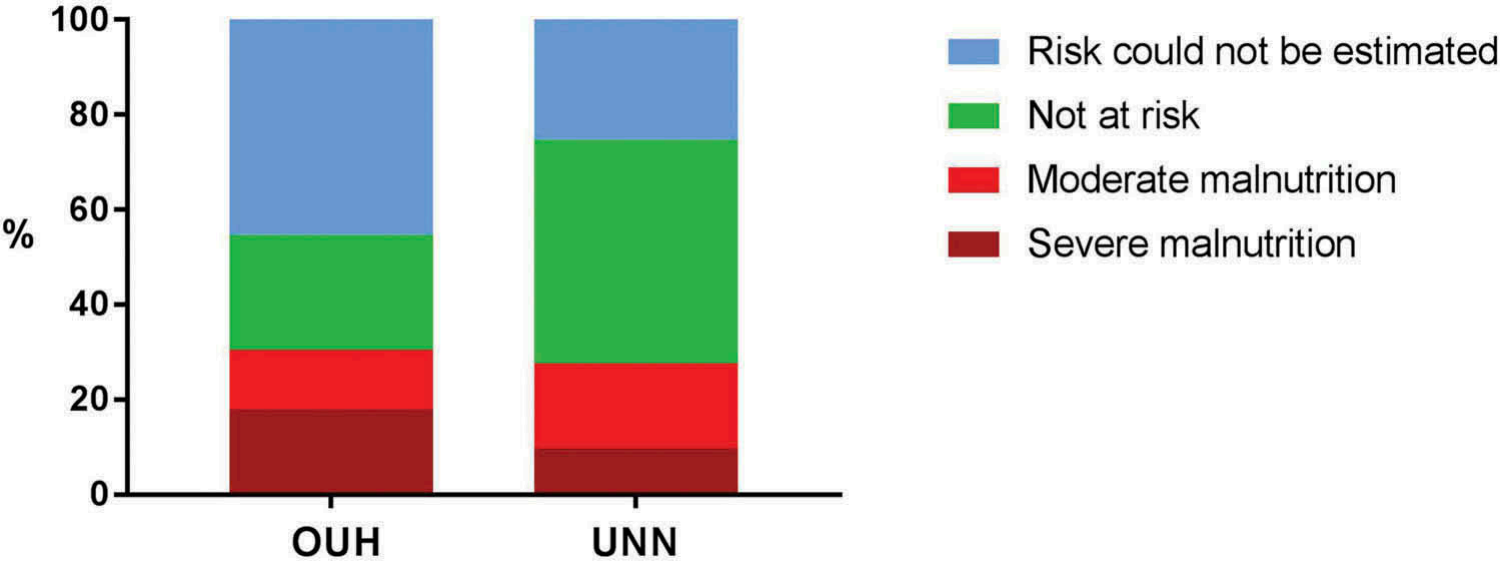
Proportions of patients with malnutrition in the two hospitals. OUH: Oslo University Hospital. UNN: University Hospital of Northern Norway (*n *= 437).

Using the Norwegian diagnostic criteria for malnutrition, malnutrition was diagnosed in 30% of the patients (Figure 2), ranging from 19 to 40% between the specialty units, oncology units having the highest rate (Figure 1). There were no differences in the overall malnutrition rate between the hospitals, but a significantly higher proportion of patients were seriously malnourished at OUH vs. UNN (18 vs. 10%, *p* < 0.001). The diagnosis could not be estimated in all patients because of missing data (Figure 2).

The estimated malnutrition rate was only 11% when calculation was based on the ESPEN consensus criteria, and did not vary much between the units (Figure 1(f)). As fat free mass was not measured, neither routinely nor at nutritionDay, the ESPEN definition could not be fully used.

Patients with a low intake at the recorded meal on nutritionDay had higher odds ratio of getting malnutrition diagnosed according to the national criteria (OR: 3.21, 95% CI1.47‒6.99 for lowest vs. normal food intake).

#### Specialty units

Oncology units had the highest frequency of patients with low BMI, and the highest weight loss and overall malnutrition rate. Surgery patients had the lowest food intake the previous week, and geriatric units had the lowest food intake at a main meal on nutritionDay.

#### Advanced nutrition treatment

Only 41% of the malnourished patients received nutritional treatment (other than hospital diet or special diet), the most frequent treatment being protein-energy rich drinks (sip-feeds) and parenteral nutrition (Table 4). On the other hand, nutritional treatment was provided to 22% of well-nourished patients and 20% of patients whose nutritional risk could not be estimated because of missing data. Significantly more frequent use of enteral nutrition and protein-energy rich drinks was reported in OUH than in UNN (Table 4).

**Table 4.** Nutrition treatments.

	Total *n*=437	OUH *n*=315	UNN *n*=122	p-value
Malnourished patients (*n*)	129	96	33	
Nutrition treatment (*n*, %)				
Any:	53 (41.1)	34 (35.4)	19 (57.6)	0.026
– Enteral nutrition	10 (7.8)	4 (4.2)	6 (18.2)	0.009
– Parenteral nutrition	24 (18.6)	16 (16.7)	8 (24.2)	ns
– Enteral + parenteral	3 (2.3)	3 (4)	0	ns
– Protein/energy drink	25 (19.4)	14 (14.6)	11 (33.3)	0.019

OUH: Oslo University Hospital; UNN: University Hospital of North Norway

### Potential cost savings

Under the assumption that the associations between malnutrition and length of stay are causal, potential cost savings of nutritional treatment can be estimated. The calculations are based on the finding that 60% of the malnourished patients in our hospitals did not receive nutritional treatment, and assuming that these patients by appropriate treatment will reduce length of stay by one day [9]. According to national statistics, there are 804,308 hospital admittances in somatic specialties in Norway each year, and the mean length of stay is 4.4 days [19]. The costs per hospital day are 1,857 euros [19]. Assuming a reduced length of stay for 18% of the patients (60% of the 30% who are malnourished, i.e. 144,774 patients) by one day, this will result in a yearly cost saving of 268,848,000 euros. To initiate and monitor the nutrition therapy, clinical dietitians have to be involved. We assume that a dietitian can treat 600 patients each year, giving a total of 241 new positions at a total cost of 15,810,000 euros. The extra cost of nutrition treatment (e.g. sip-feeding) is only 5 euros per patient each day, giving a total sum of 2,896,000 euros per year. To summarize, this will be a yearly cost saving of 250,141,000 euros (Table 5).

**Table 5.** Potential cost savings.

	Costs in EURO^a^
Costs of one extra day in hospital for 60% of malnourished patients (*n*=144 775)	268 847 000,-
Costs of nutrition treatment in hospital:	18 706 000,-
Sip-feed for 4 days^b^	2 896 000,-
Dietitians^c^	15 810 000,-
Total potential cost savings	250 141 000.-

^a^ Rounded to nearest 1000 ^b^8 sip-feeds will cost 20 euros/patient ^c^ Costs of 241 new positions for dietitians

## Discussion

### Summary of results

In this point-prevalence study of malnutrition in Norwegian hospitals, a high proportion of malnourished patients did not receive adequate nutritional treatment, indicating a potential for improved nutritional care and cost savings. Involuntary weight loss, as well as low food intake before and during hospital stay was widespread at both hospitals. Patients at oncology units had the lowest BMI, and the highest weight loss and malnutrition-rate, which needs further attention. Surgery patients had the lowest food intake in the previous week, and geriatric units had the lowest food intake on NutritionDay

### Clinical implications

We found that the malnutrition rate was 30% using the national guidelines for diagnosis of malnutrition. This estimate was in line with previous studies from European hospitals using different screening tools for assessing nutritional risk [7]. We expected the malnutrition rate to be lower than the nutritional risk rate, but the similar numbers might indicate that most patients at risk are also malnourished. As malnutrition is associated with more frequent readmissions, greater morbidity and mortality, this may have several clinical and economic consequences if the association is causal [6,8,20,21].

As many as 59% of the malnourished patients did not receive any advanced nutritional treatment. This indicates that nutritional therapy is not provided to all patients in need. One explanation might be that the patients are not screened according to national guidelines, or that the risk screening does not lead to appropriate action. Our results confirm the finding from an earlier Norwegian study, reporting that only 53% of the at-risk patients received nutritional treatment and 5% were referred to a dietitian [22]. On the other hand, 22% of the well-nourished patients received nutritional therapy. This might be patients receiving enteral feeding after surgery, because of dysphagia or other patients identified at nutritional risk.

The ESPEN criteria for diagnosis of malnutrition gave a lower estimate of malnourished patients compared to the usual diagnostic criteria, and this is in accordance with other publications [5,6]. The main differences between our national and the ESPEN criteria for diagnosis of malnutrition are that data on food intake are included in the national definition and that the combination of weight loss and reduced fat free mass is an option in the ESPEN definition. However, fat-free mass was not measured at nutritionDay, and the ESPEN criteria could not be fully used in our present study. This may also be the case in other hospitals, and this is a limitation with the ESPEN criteria. Nevertheless, the malnutrition rate may also be over-estimated with the ESPEN criteria because patients should be considered ‘at risk of malnutrition’ by any validated risk screening tool prior to diagnosis, and no such risk assessment was done in the present study.

Data on weight or length were frequently missing in the patient records, and BMI could only be calculated in two-thirds of the patients on nutritionDay. Both weight loss and BMI are independently associated with morbidity and mortality [8], and failure to monitor these parameters may delay initiation of nutritional treatment. It must be noted that low BMI (<18.5 kg/m^2^) was present in few of the patients, indicating that this parameter alone will not detect all malnourished patients. This commonly used cut-off point might also be too low, as BMI less than 23 kg/m^2^ is associated with increased mortality in older adults [23]. Thus, monitoring of nutritional risk and further assessment seems necessary, as recommended by ESPEN and national authorities [13].

We found that the proportion of patients with very low food intake (eating less than a quarter of the meal at nutrionDay) was high. The number varied from 25 to 55%, oncology units having the highest frequency. Earlier nutritionDay surveys showed that low food intake was an independent risk factor for length of stay and mortality, also after controlling for several possible confounders [18,20]. It is still not known whether low food intake has a causal effect or is a marker of severe disease. Both explanations could be true because malnutrition can be both a cause and a consequence of disease. Close monitoring of food intake during hospital admittance seems warranted, especially in oncology, surgery and geriatric units. Rapid, valid and easy-to-use tools should be developed to implement monitoring of food intake as a daily routine.

### Potential cost savings

Nearly 60% of the malnourished patients in Norway did not receive nutritional treatment, indicating a potential for improved nutritional care and cost savings.

We estimated the potential cost savings to be in the order of 250 million euros per year. Although this number is more than twice as high as earlier Norwegian calculations [11,12], this is a conservative estimate. We based our calculations on a reduction in length of stay of only one day, but a recent report from the Netherlands showed by regression analyses that being at risk of malnutrition was associated with 1.4 days longer stay [9]. A previous Norwegian study showed a difference of 3.5 days of stay between patients at risk vs. patients not at risk for malnutrition [8], thus the real cost-savings may be even higher than shown in our calculations. Furthermore, the extra costs of treatment, both the oral feeding and the costs of dietitians are included in our calculations.

Our findings are in agreement with Elia [24], who conducted a systematic review to assess whether oral nutritional supplements (ONS) could produce cost savings and cost-effective outcomes. The results showed significant cost savings of ONS compared to the control groups (median cost saving 12.2%; *p* < 0.01). Cost savings were typically associated with significantly reduced mortality, complication rate, shorter length of stay and fewer pressure ulcers. Most of the studies in this systematic review were retrospective analyses of randomized controlled trials. Although this may implicate a causal association between malnutrition and clinical outcomes, there is a need for more prospective studies designed to examine primary economic outcomes.

### Methodological considerations

A strength of the present study is that the nutritionDay forms and methodology are used worldwide, giving results that can be compared to other countries and over time.

A limitation with this study is the representativeness. The units were recruited on a voluntary basis and this has probably resulted in participation from the units where the healthcare professionals had the most interest or knowledge in nutrition care. If so, the number of malnourished patient who did not receive treatment might be even higher than reported here. The selection bias might be higher in UNN were only six units agreed to participate. However, the participating units represented a wide range of specialties, and all results were pointing in the same direction. Moreover, recruitment of patients within each unit was good, increasing the validity of data for each unit. Two hospitals from different regions participated, and ideally, hospitals from all four regions should be included to achieve country representative data.

## Conclusions

In this point-prevalence study of malnutrition in Norwegian hospitals, six out of 10malnourished patients did not receive nutritional treatment. The malnutrition rate was 30% using the national guidelines compared to 11% when the new ESPEN consensus criteria were used.

Data on weight or length were frequently missing in the patient records, and BMI could only be calculated in two-thirds3 of the patients. The frequency of low BMI (<18.5 kg/m^2^) was only 5% indicating that routine weighing will not detect all malnourished patients.

Involuntary weight loss and sub-normal food intake were widespread and confirm the need for nutritional screening as recommended. Reduced food intake may be an indication of malnutrition, and we suggest that monitoring of food intake should be included as a routine for all patients at risk of malnutrition.

All indicators of malnutrition in the two University hospitals in Norway were in line with previous reports from nutritionDay in other countries. There is a great potential for improved nutritional care and cost savings.
